# Innate and Adaptive Immune Responses Both Contribute to Pathological CD4 T Cell Activation in HIV-1 Infected Ugandans

**DOI:** 10.1371/journal.pone.0018779

**Published:** 2011-04-19

**Authors:** Michael A. Eller, Kim G. Blom, Veronica D. Gonzalez, Leigh Anne Eller, Prossy Naluyima, Oliver Laeyendecker, Thomas C. Quinn, Noah Kiwanuka, David Serwadda, Nelson K. Sewankambo, Boonrat Tasseneetrithep, Maria J. Wawer, Ronald H. Gray, Mary A. Marovich, Nelson L. Michael, Mark S. de Souza, Fred Wabwire-Mangen, Merlin L. Robb, Jeffrey R. Currier, Johan K. Sandberg

**Affiliations:** 1 Makerere University Walter Reed Project, Kampala, Uganda; 2 U. S. Military HIV Research Program, Rockville, Maryland, United States of America; 3 Center for Infectious Medicine, Department of Medicine, Karolinska Institutet, Karolinska University Hospital Huddinge, Stockholm, Sweden; 4 National Institutes of Allergy and Infectious Diseases, National Institutes of Health, Bethesda, Maryland, United States of America; 5 Johns Hopkins School of Medicine, Baltimore, Maryland, United States of America; 6 School of Public Health, Makerere University College of Health Sciences, Kampala, Uganda; 7 Rakai Health Sciences Program, Uganda Virus Research Institute, Entebbe, Uganda; 8 Faculty of Medicine, Makerere University College of Health Sciences, Kampala, Uganda; 9 Faculty of Medicine Siriraj Hospital, Mahidol University, Bangkok, Thailand; 10 Columbia University Mailman School of Public Health, New York, New York, United States of America; 11 Johns Hopkins Center for Global Health, Baltimore, Maryland, United States of America; 12 Armed Forces Research Institute of Medical Sciences, Bangkok, Thailand; University of California San Francisco, United States of America

## Abstract

HIV-1 disease progression is associated with persistent immune activation. However, the nature of this association is incompletely understood. Here, we investigated immune activation in the CD4 T cell compartment of chronically HIV-1 infected individuals from Rakai, Uganda. Levels of CD4 T cell activation, assessed as co-expression of PD-1, CD38 and HLA-DR, correlated directly to viral load and inversely to CD4 count. Deeper characterization of these cells indicated an effector memory phenotype with relatively frequent expression of Ki67 despite their PD-1 expression, and levels of these cells were inversely associated with FoxP3+ regulatory T cells. We therefore use the term deregulated effector memory (DEM) cells to describe them. CD4 T cells with a DEM phenotype could be generated by antigen stimulation of recall responses *in vitro*. Responses against HIV-1 and CMV antigens were enriched among the DEM CD4 T cells in patients, and the diverse Vβ repertoire of DEM CD4 T cells suggested they include diverse antigen-specificities. Furthermore, the levels of DEM CD4 T cells correlated directly to soluble CD14 (sCD14) and IL-6, markers of innate immune activation, in plasma. The size of the activated DEM CD4 T cell subset was predictive of the rate of disease progression, whereas IL-6 was only weakly predictive and sCD14 was not predictive. Taken together, these results are consistent with a model where systemic innate immune activation and chronic antigen stimulation of adaptive T cell responses both play important roles in driving pathological CD4 T cell immune activation in HIV-1 disease.

## Introduction

HIV-1 disease progression is associated with chronic activation of the immune system [1],[2],[3],[4],[5],[6],[7],[8] (reviewed in [9], [10]), but the mechanisms involved remain incompletely understood. Unraveling the causes, interactions, and mechanisms underlying immune activation is thus central to the understanding of HIV pathogenesis and one of the major “roadblocks” in HIV research [11]. One proposed cause of systemic immune activation is microbial product translocation across a gut barrier damaged by intense HIV replication [12], [13]. Brenchley et al. found that lipopolysaccharide (LPS) and soluble CD14 (sCD14), thought to be correlative markers of microbial translocation, in plasma were associated with CD8 T cell activation in chronic HIV-1 infection [12]. A role for microbial translocation is supported by findings in natural SIV infection of African green monkeys and sooty mangabeys [14], [15], [16]. However, high levels of LPS in rhesus monkeys may not predict faster disease progression [17]. A second proposed cause behind chronic activation is direct innate recognition of viral RNA and subsequent activation of the type I interferon (IFN) response, and this is also supported by data from natural hosts of SIV, which exhibit a lower and less sustained response to viral RNA [18], [19], [20]. Interestingly, gene-expression profiling of monocytes from HIV-1 infected patients suggested that IFNα may be an important factor in the activation of these cells [21]. A third possible cause of activation in the T cell compartment is direct TCR-mediated recognition of antigens, and fourth possibility is bystander activation, which may be driven by the aforementioned mechanisms or have other causes [22]. It is important to recognize that these four different suggested mechanisms are not mutually exclusive.

Immune cell exhaustion plays a role in loss of control of HIV-1 viremia. Programmed death 1 (PD-1, or CD279), is a negative regulator of T cell activation [23]. The association between PD-1 expression and poor function of HIV-specific T cells and aspects of HIV disease has been observed for CD8 T cells [24], [25], [26], [27], and for CD4 T cells [28]. Blocking of the PD-1 pathway *in vivo* in macaques results in increased SIV-specific T cell function and longer survival of the animals [29], [30]. In summary, uncontrolled activation combined with exhaustion and excessive negative feedback regulation contribute to a situation with poor immune control of HIV.

FoxP3+ regulatory T cells (Tregs) are important for maintaining immune tolerance and for avoiding inappropriate immune activation and autoimmunity [31]. In HIV-1 infection, Tregs probably have a beneficial function by limiting the damage of immune activation [32], [33], [34], [35]. However, loss of Tregs from blood may represent redistribution to lymphoid tissues where they might interfere with T cell control of HIV-1 replication [36], [37]. The beneficial role of Tregs in HIV-1 disease is thus not entirely clear-cut. In this study, we investigated aspects of immune activation and immune regulation with the aim to better understand pathological immune activation in the CD4 T cell compartment of HIV-1 infected patients in sub-Saharan Africa, and to better understand relationships between CD4 T cell activation and possible causes of such activation.

## Materials and Methods

### Study cohort and samples

Study participants aged 15–49 years were enrolled in a prospective community-based cohort to assess the prevalence and incidence of HIV-1 infection in Rakai District, Uganda, from 1998 until 2004 (Table 1) [38], [39], [40]. Infected subjects were identified between 1997 and 2002 with continued annual follow up through 2008. The study was approved by institutional review boards in the US and Uganda: The institutional Review Boards of Uganda's National Council for Science and Technology (UNCST) and the Uganda Virus Research Institute’s Science and Ethics Committee, as well as the Division of Human Subjects Protection at the Walter Reed Army Institute of Research. Written informed consent given by all participants, and written informed consent was obtained from the parent or legal guardian of those aged 17. PBMC samples were isolated and cryopreserved as described [41], from 103 randomly selected HIV-1 sero-positive individuals and 40 community-matched sero-negative controls. No patients were on antiretroviral therapy. HIV-1 testing was performed as described [40]. Positive samples were subjected to the Amplicor HIV-1 Monitor test, version 1.5 (Roche Diagnostics, Indianapolis, IN).

**Table 1:** Study population descriptive statistics.

	HIV-1 negative	HIV-1 positive
Subjects, no.	40	103
Female, no. (%)	20 (50%)	65 (63%)
Male, no. (%)	20 (50%)	38 (37%)
Age, median years (range)	29 (20–42)	30 (17–53)
Time from sero-conversion, median days (range)	NA	913 (22–2284)
Viral load, median copies/ml (range)	NA	35,828 (528–1,685,638
CD4 absolute count, median cells/µl (range)	NA	513 (1–1385)
HIV-1 subtype A, no. (%)	NA	35 (34%)
HIV-1 subtype D, no. (%)	NA	68 (66%)
Hepatitis B virus, no. (%)	3 (8%)	8 (8%)
Syphilis, no. (%)	3 (8%)	9 (9%)

NA indicates not applicable.

### Flow cytometry and mAbs

Cryopreserved specimens were thawed and washed, and counts and viability assessed on the Guava PCA (Guava Technologies, Hayward, CA), using Guava ViaCount reagent. Cells washed with PBS/BSA buffer, and stained at 4°C for 30 min in 96-well V-bottom plates in the dark. mAbs used in flow cytometry; anti-HLA-DR FITC or V450, IgG2a FITC, anti-PD-1 PE or IgG1 PE, anti-CD3 PerCP-Cy5.5 or APC-H7, anti-CCR7 PE-Cy7, anti-CD8 PE-Cy7 or PE-TR or Qdot605, anti-CD38 APC or IgG1 APC, anti-CD4 Pacific Blue (PB) (all from BD Biosciences, San Jose, CA), and Aqua Live/Dead Stain (Invitrogen, Carlsbad, CA). The anti-PD-1 PE (clone EH12) was a kind gift from Maria Jaimes at BD Biosciences. Anti-CD19 PE-Cy5, and anti-CD45RO eF650NC were from eBioscience (San Diego, CA), and anti-CD28 PerCP-Cy5.5 was from BioLegend (San Diego, CA). Anti-CD4 Qd605, and anti-CD14 PE-Cy5 were from Invitrogen. Anti-CD4 ECD was from Beckman Coulter (Brea, CA). For assessment of Ki67 expression, cells were fixed and permeabilized with eBioscience fix/perm for 60 min at 4°C and stained with anti-Ki67 FITC (BD Bioscience) for 30 min. Tregs were identified using anti-CD25 PE, anti-CD3 PerCP-Cy5.5, anti-CD127 APC, and anti-CD4 PB (all from BD Biosciences). Samples were washed, permeabilized and fixed using a kit optimized for FoxP3 staining (eBioscience, San Diego, CA), and stained with anti-FoxP3 Alexa Fluor 488 (BioLegend San Diego, CA). Flow cytometry data was acquired using a BD LSR II instrument or a BD FACS Canto II instrument (BD Biosciences). Lymphocyte immunophenotyping was performed using FACS MultiSET System and run on a FACSCalibur using the single platform Multi-test 4-color reagent in combination with TruCount tubes (BD Biosciences).

IL-1β, IL-12p70, TNFα, IL-10, IL-6 and IL-8 were measured in plasma using a Human Inflammatory Cytokine Bead Array (BD Biosciences), and a FACSCalibur flow cytometer. sCD14 was measured in plasma using a quantitative sandwich enzyme immunoassay (R&D Systems, Minneapolis, MN). Absorbance was measured using a BioTek ELx800 plate reader and analyzed using the KC4 data analysis software (BioTek, Winooski, VT).

To examine Vβ distribution of T cells, cryopreserved PBMC specimens were thawed and stained for HLA-DR, PD-1, CD3, CD38, CD4, CD8 and Aqua Live/Dead. Anti-TCR Vβs included mAbs against Vβ1, Vβ2, Vβ3, Vβ5.1, Vβ5.2, Vβ7, Vβ8, Vβ11, Vβ12, Vβ13.1, Vβ13.6, Vβ14, Vβ16, Vβ17, Vβ20, Vβ21.3, and Vβ22 conjugated to FITC (all from Beckman Coulter). After incubation, samples were washed 2 times and fixed in 2% formaldehyde before acquisition.

### Functional assays

To assess the generation of immune activation *in vitro*, PBMC from HIV-1 sero-negative donors were thawed and 1×10^5^ cells per well incubated in 96-well U-bottom plates with Adenovirus serotype 5 (Ad5) hexon peptides (Miltenyi Biotec Inc., Auburn, CA) at 1 µg/ml, *Candida albicans* allergenic extract (Greer laboratories Inc., Lenoir, NC) at a 1∶500 dilution, Cytomegalovirus (CMV) viral lysate (Advanced Biotechnologies Inc., Columbia, MD) at 0.5 µg/ml, CMVpp65 PepMix (JPT Peptide Technologies GmbH, Berlin, Germany) at 1 µg/ml, Concanavalin-A (ConA) (Sigma, St. Louis, MO) at 1.25 µg/ml, Phytohemagglutinin (PHA) (Sigma) at 2 µg/ml, Pokeweed Mitogen (PWM) (Sigma) at 20 µg/ml, and Staphylococcal Enterotoxin B (SEB) (Sigma) at 10 ng/ml for 3-6 days. Supernatants were collected and cells were stained for expression of CD38, HLA-DR, and PD-1. To assess antigen-specific CD4 T cell responses, PBMC were thawed and cultured overnight (12 hrs) with HIV-1 subtype A Gag peptides (1 µg/ml), HIV-1 subtype A whole inactivated virus (HIV_WIV_) (Lot P4039) and microvesicles (Lot P3772) at 500 ng/ml (courtesy of Jeff Lifson (NCI Frederick, MD), CMVpp65 peptides, CMV lysate, or SEB. Brefeldin A (Sigma, St. Louis, MO) was present during the final 6 hrs of incubation. After incubation, samples were fixed, washed, and permeabilized using BD PERM Wash (BD Biosciences), and stained with anti-IFNγ PB (eBiosciences) and anti-TNFα PE-Cy7 (BD Biosciences) before acquisition on an BD LSRII cytometer (BD Biosciences).

### Statistical analysis

Cytometry analysis was performed using FlowJo version 8.5 (Tree Star, Ashland, OR). All statistical analysis was performed using Graph Pad Prism version 5.0a for Mac OSX (GraphPad Software, La Jolla, CA). Comparisons between groups were performed using Fisher's exact test for categorical data. Direct comparisons between two groups were performed using the non-parametric Mann-Whitney *U* test. Associations between groups were determined by Spearman’s rank correlation. Kaplan-Meier survival curves were calculated from the estimated time of infection to reaching CD4 counts<250 cells/µl of whole blood. For paired observations a paired t test was used. P values<0.05 were considered statistically significant. Statistical methods and analyses were reviewed by EmpiriStat, Inc. (Mount Airy, MD).

## Results

### Activated CD4 T cells with co-ordinate expression of HLA-DR, CD38, and PD-1 are deregulated effector memory cells and elevated in chronically HIV-1 infected patients in Rakai, Uganda

**Figure 1:**
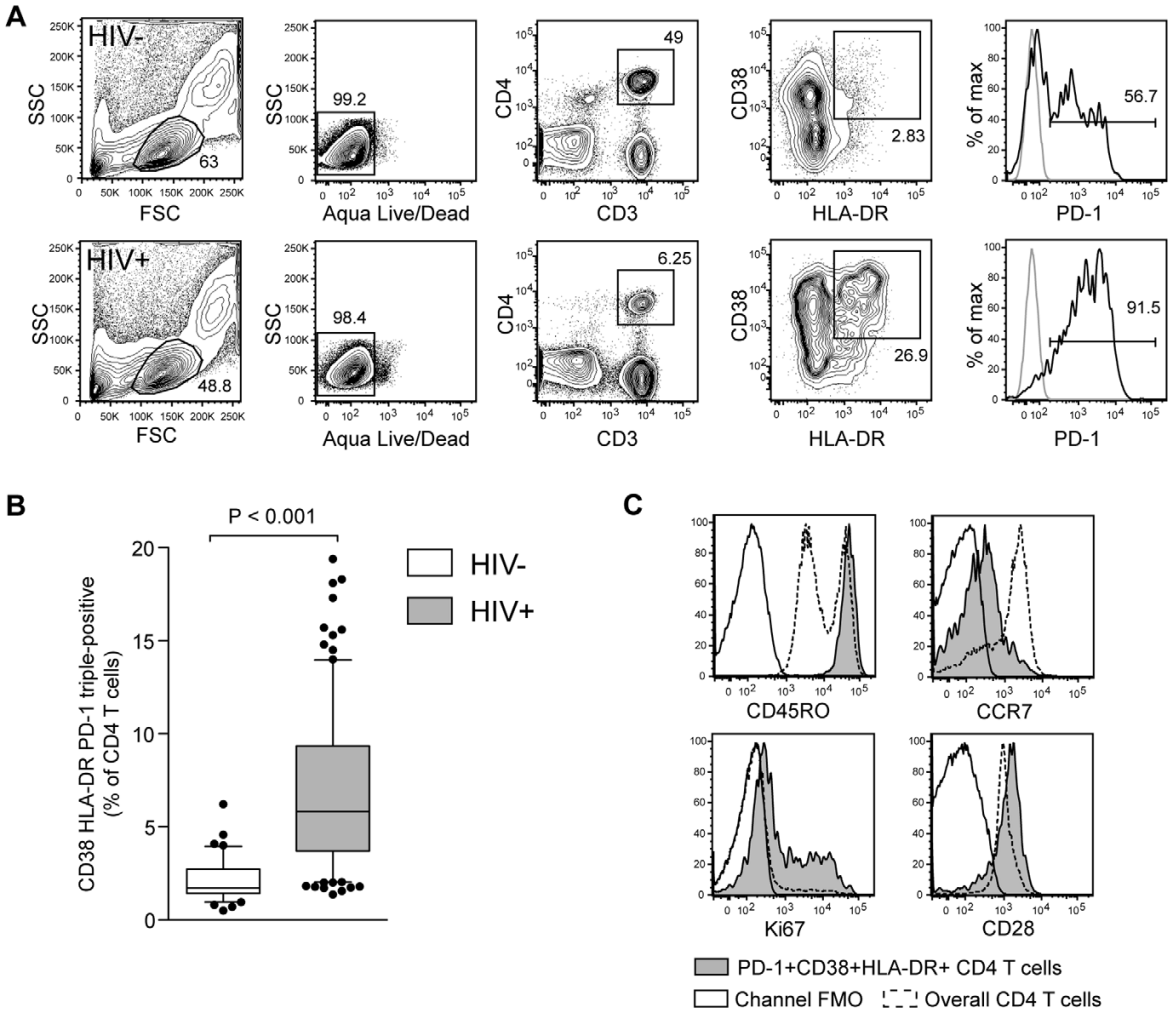
PD-1, HLA-DR, and CD38 together identify an effector memory CD4 T cell subset elevated in HIV-1 infection. (A) Identification of CD4 T cells co-expressing PD-1, HLA-DR and CD38 in HIV-1 infected and uninfected subjects. (B) Box and whisker plots showing the median and 10–90 percentile percentage of CD38, HLA-DR and PD-1 triple-expression in CD4 T cells in HIV negative (n = 40) and HIV positive (n = 103) subjects. (C) In order to assess the phenotype of the activated CD4 T cells, PBMC were analyzed by flow cytometry for the expression of CD45RO, CCR7, CD28, and Ki67. Filled line represents CD4 cells triple-positive for CD38, HLA-DR, and PD-1, while dashed line represents data from the overall CD4 compartment and empty filled line is the fluorescence minus one (FMO) control for that channel.

HIV-1 negative (n = 40) and HIV-1 positive (n = 103) individuals from a cohort in Rakai, Uganda, were chosen to study CD4 T cell activation in untreated HIV-1 infection (Table 1). Activated CD4 T cells expressing CD38 and HLA-DR were identified by flow cytometry (Figure 1A). These cells were largely positive for PD-1, suggesting an exhausted phenotype. The CD38+HLA−DR+PD-1+ CD4 T cells were significantly elevated in HIV-1 infected patients as compared to healthy donors (P<0.001) (Figure 1B). To further characterize the PD-1+HLA-DR+CD38+ CD4 T cell subset, we analyzed their expression of CD45RO, CCR7, CD28, and Ki67. These cells were largely CD45RO+, CD28+, and negative or dim for CCR7. In addition, a proportion expressed the proliferation marker Ki67 (Figure 1C). This expression pattern is consistent with an effector memory profile that retains the proliferative capacity, despite the expression of PD-1.

**Figure 2:**
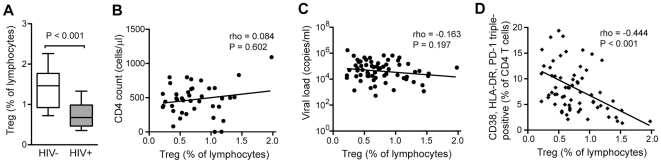
Inverse correlation between FoxP3+ regulatory T cells and PD-1+CD38+HLA-DR+ CD4 T cells. (A) Box and whisker plots showing the median and 10–90 percentile percentage of FoxP3+CD25+CD127- regulatory CD4 T cells in HIV negative and HIV positive subjects. (B) Spearman rank correlation between regulatory CD4 T cells and CD4 absolute T cell counts. (C) Spearman rank correlation between regulatory CD4 T cells and viral load. (D) Spearman rank correlation between regulatory CD4 T cells and CD38, HLA-DR and PD-1 triple-positive CD4 T cells in blood.

Regulatory activity mediated by Tregs is important to limit inappropriate or persistent immune activation [31]. The CD4+CD25+CD127-FoxP3+ Treg compartment was reduced in HIV-1 infected subjects (Figure 2A), but the Treg levels did not correlate with the CD4 count (Figure 2B), or viral load (Figure 2C). However, there was an inverse relationship between FoxP3+ Tregs and PD-1+HLA-DR+CD38+ CD4 T cells (r = −0.444, P<0.001) (Figure 2D). The Ki67+ effector memory phenotype of the PD-1+HLA-DR+CD38+ CD4 T cells, and the association between these cells and the decline in the Treg compartment, together supports a model in which these cells are deregulated effector memory (DEM) cells.

### Correlates between DEM CD4 T cell expansion and HIV-1 disease

**Figure 3:**
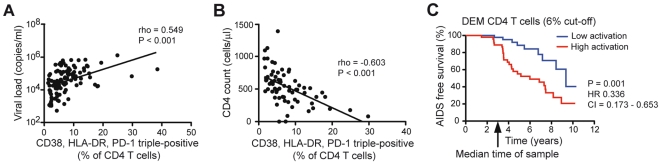
Levels of deregulated effector memory CD4 T cells correlate to and predict HIV-1 disease progression. (A) Spearman rank correlation between percentages of CD38, HLA-DR and PD-1 triple-expressing CD4 T cells and HIV-1 plasma viral load. (B) Spearman rank correlations between percentages of CD38, HLA-DR and PD-1 triple-expressing CD4 T cells and CD4 counts. (C) Kaplan-Meier analysis comparing the time to AIDS, as defined by CD4 T cell counts less than 250 cells/µl of whole blood. HIV-1 positive subjects with CD38, HLA-DR and PD-1 triple-expressing CD4 T cells above the median (6%) were more likely to advance to AIDS (Log-rank  = 0.001, Hazard Ratio (HR) 0.336, 95% CI = 0.173 to 0.653).

We next investigated associations between immune activation in CD4 T cells and aspects of HIV-1 disease in this cohort. The size of the DEM CD4 T cell subset was directly correlated with viral load (r = 0.549, P<0.001) (Figure 3A), and inversely correlated with CD4 count (r = −0.603, P<0.001) (Figure 3B). Next, the ability to predict progression to AIDS in this cohort using the DEM CD4 T cell phenotype was assessed. AIDS was defined as CD4 counts below 250 cells/µl. Patients were divided into two groups for having levels of DEM CD4 T cells above or below the median (6%) (Figure 3C). Values below the median overlapped with the range observed in uninfected subjects. Patients with levels above 6% of CD4 T cells were more likely to progress to AIDS (log-rank P = 0.001, HR 0.336, 95% CI = 0.173–0.653). Notably, the two groups with high and low levels of DEM CD4 T cells did not differ significantly in their estimated time since infection (data not shown). Thus, the DEM phenotype identifies a state of pathological immune activation in the CD4 T cell compartment which is associated with HIV-1 disease progression.

### Associations between measures of HIV disease progression and soluble CD14 and IL-6, measures of innate immune activation, in plasma

**Figure 4:**
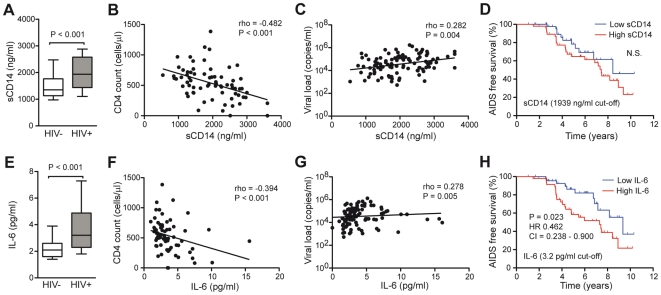
Associations between sCD14 and IL-6 levels in plasma and measures of HIV-1 disease progression. (A) Box and whisker plot showing sCD14 levels in plasma from HIV-1 infected (n = 103) and HIV-1 uninfected (n = 40) subjects. Spearman rank correlations between sCD14 in plasma and (B) CD4 absolute T cells counts, and (C) viral load. (D) Kaplan-Meier analysis comparing the time to AIDS. HIV-1 positive subjects with sCD14 levels above and below the median (1939 ng/ml) were not statistically different (Log-rank P = 0.185, Hazard Ratio (HR) 0.631, 95% CI = 0.319 to 1.247). (E) Box and whisker plot showing IL-6 levels in plasma from HIV-1 infected (n = 103) and HIV-1 uninfected (n = 40) subjects. Spearman rank correlations between IL-6 in plasma and (F) CD4 absolute T cells counts and (G) viral load. (H) Kaplan-Meier analysis comparing the time to AIDS. HIV-1 positive subjects with IL-6 levels above the median (3.2 pg/ml) more likely to advance to AIDS (Log-rank P = 0.023, HR 0.462, 95% CI = 0.238–0.900).

CD14 is released from monocytes upon activation, and soluble CD14 (sCD14) in plasma can be used as a marker of such activation in response to innate stimuli [12], [42]. sCD14 was elevated in HIV-1 infected subjects as compared to uninfected controls (P<0.001) (Figure 4A), and CD4 counts in infected subjects correlated inversely with sCD14 in plasma (r = −0.482, P<0.001) (Figure 4B). There was also a weak positive correlation between sCD14 levels and viral load (r = 0.282, P = 0.004) (Figure 4C). Importantly, however, subjects with high and low sCD14 levels did not differ in their AIDS-free survival during clinical follow-up (Figure 4D). Similarly, patients with Treg levels above or below the median percentage did not differ in their AIDS-free survival (data not shown).

IL-1β, IL-12p70, TNFα, IL-10, IL-6 and IL-8 were measured in plasma from HIV-1 infected and uninfected subjects, and IL-6 and IL-8 were found to be significantly elevated in the infected group (Figure 4E and data not shown). However, only IL-6 showed an inverse correlation with CD4 counts (r = −0.394, P<0.001) (Figure 4F), and a weak positive association with viral load (r = 0.278, P = 0.005) (Figure 4G). In contrast to the pattern with sCD14, having above the median level of IL-6 in plasma was a significant predictor of faster disease progression (log-rank P = 0.023, HR 0.462, 95% CI = 0.238-0.900) (Figure 4H). In summary, measures of innate immune activation, sCD14 and IL-6, show a degree of association with HIV-1 disease. However, these associations are weaker than those observed between CD4 count, viral load, AIDS-free survival, and the size of the DEM CD4 T cell population.

### Analysis of relationships between the DEM CD4 T cell expansion and measures of innate immune activation

**Figure 5:**
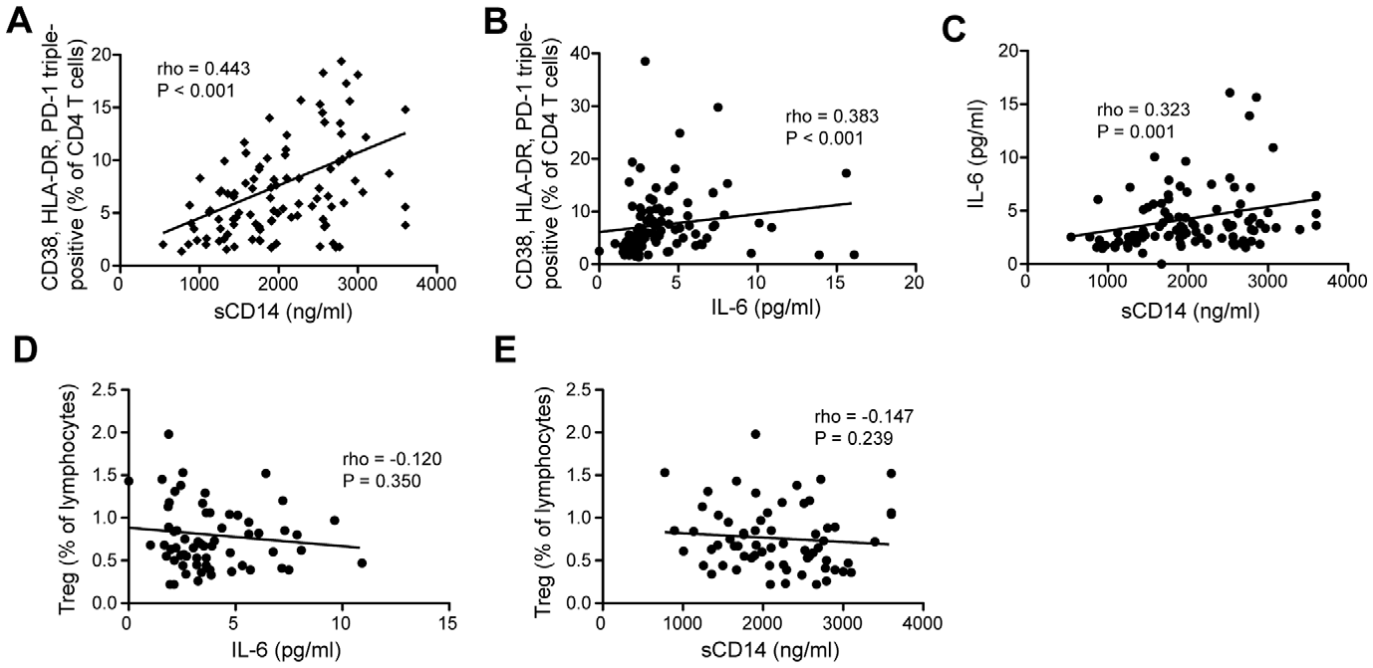
sCD14 and IL-6 correlate with levels of deregulated effector memory CD4 T cells, but not with Tregs. (A) Spearman rank correlation between sCD14 in plasma and CD38, HLA-DR and PD-1 triple-positive CD4 T cells in blood. (B) Spearman rank correlation between IL-6 in plasma and CD38, HLA-DR and PD-1 triple-positive CD4 T cells. (C) Spearman rank correlation between IL-6 and sCD14 in plasma. (D) Spearman rank correlation between Tregs and IL-6. (E) Spearman rank correlation between Tregs and sCD14 levels.

These data behooved us to next search for association between the DEM CD4 T cell population, Tregs, sCD14 levels, and IL-6 levels. Interestingly, the size of the DEM CD4 T cell population was directly associated with levels of sCD14 (r = 0.443, P<0.001) (Figure 5A), and levels of IL-6 (r = 0.383, P<0.001) (Figure 5B). The two measures of innate immune activation, IL-6 and sCD14, were weakly correlated (r = 0.323, P = 0.001) (Figure 5C). Tregs showed an inverse correlation with DEM CD4 T cells (r = −0.444, P<0.001), but no significant correlation with CD4 count and viral load (Figure 2). Similarly, there was no correlation between Treg levels, and levels of sCD14 or IL-6 (Figure 5D and E, respectively). Thus, DEM CD4 T cell expansion is associated with both innate immune activation and decline in circulating Tregs. However, innate immune activation and changes in Tregs show no apparent association.

### Expansion of DEM CD4 T cells can be driven by antigen *in vitro*

**Figure 6:**
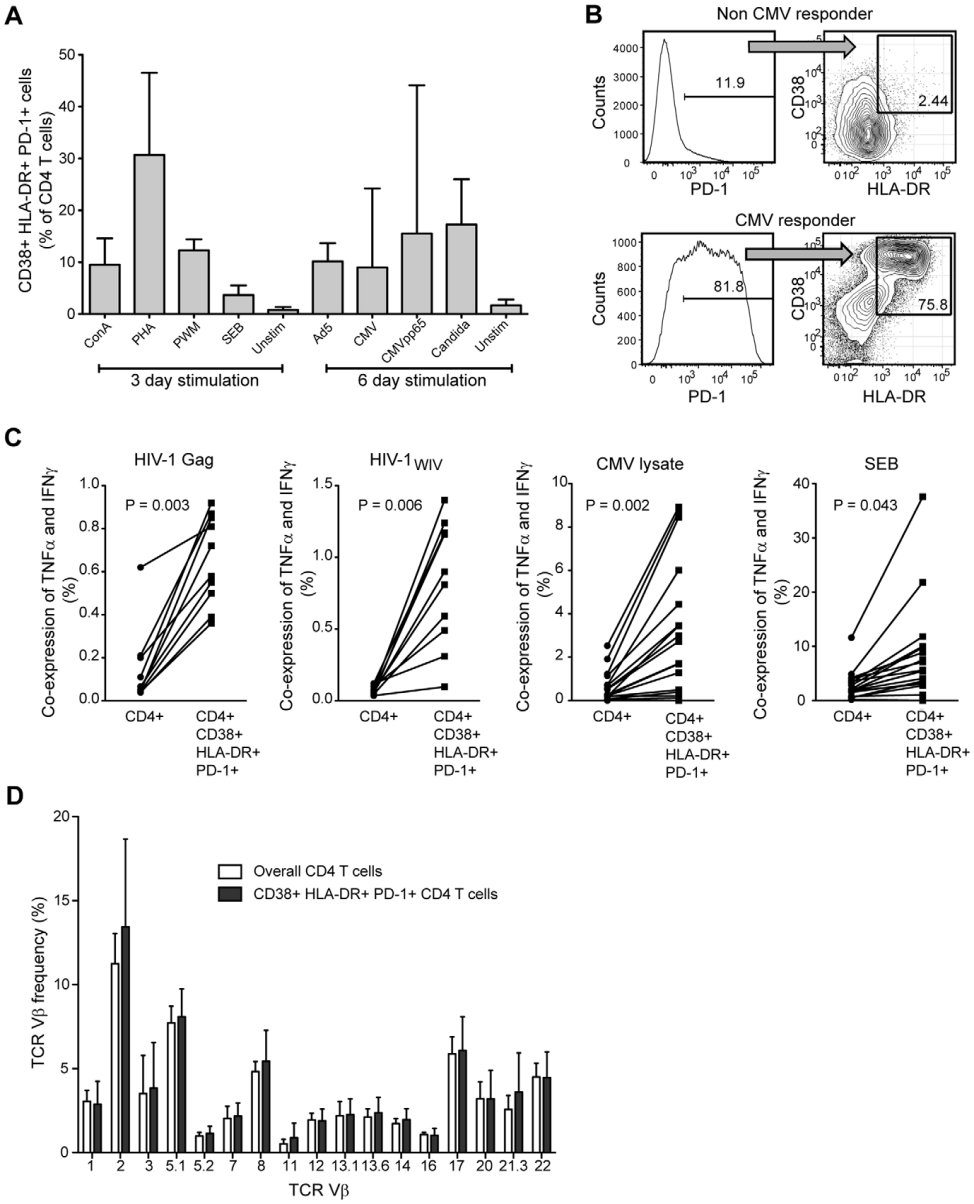
CD38+HLA-DR+PD-1+ cells can be driven by antigen *in vitro*, but display a non-biased Vβ distribution *in vivo*. (A) Experiments were conducted to assess the ability to generate the combined expression of CD38, HLA-DR, and PD-1 on CD4 T cells after three days of mitogen stimulation, or six days of antigen or superantigen stimulation of PBMC from four healthy donors. Bars represent mean and standard error of the mean. (B) Example PD-1 expression flow cytometry histogram and contour plot for CD38 and HLA-DR expression on CD4 T cells from one CMV non-responder (top panels), and one CMV responder after six days of stimulation with CMV lysate. (C) In order to characterize the prevalence of antigen specific cells in the CD38, HLA-DR and PD-1 triple-positive CD4 T cell subset, thawed PBMC were stimulated with HIV Gag peptide pool, whole inactivated HIV-1 (HIV_WIV_), CMV pp65 peptide pool and lysate, and the superantigen SEB. Grouped columnar scatter plots for responses to Gag peptides or whole inactivated virus (n = 10), CMV lysate responders (n = 18) and SEB responders (n = 18). Cells co-expressing IFNγ and TNFα were considered positive in this assay. Data were analyzed using the paired t test. (D) In order to assess the TCR Vβ repertoire of CD38+HLA-DR+PD-1+ CD4 T cells, the representation of 17 different Vβ specificities were analyzed by flow cytometry. Grouped vertical bar graph showing the mean and standard deviation for 10 HIV-1 infected patients. White bars represent the overall CD4 T cell compartment, while grey bars represent the CD38, HLA-DR, and PD-1 triple-expressing CD4 T cells. There is no statistically significant difference in representation of any Vβ between the two CD4 T cell subsets.

We next investigated factors that might be involved in driving the expansion of PD-1+HLA-DR+CD38+ CD4 T cells. To begin to address the hypothesis that high antigen loads may drive this population, we first stimulated PBMC from healthy donors with a set of different recall antigens, mitogens and superantigen (Figure 6A). Several of the stimuli, in particular CMV and Candida antigens, provoked significant expansions of triple-positive CD4 T cells (Figure 6B).

In a second approach, we assessed CD4 T cell responses in the HIV-1 infected patients against CMV, HIV and SEB (Figure 6C). Responses against all these antigens were measured by intracellular co-staining for both IFNγ and TNFα, and found to be enriched in the DEM CD4 T cells as compared to the overall CD4 compartment. These data collectively indicate that the DEM CD4 T cell phenotype may be driven by antigen stimulation.

### DEM CD4 T cells display a non-biased Vβ distribution

Enrichment of antigen-specific responses in the DEM CD4 T cells is consistent with an antigen-driven expansion of these cells. Nevertheless, the percentages of antigen-specific responses in the DEM CD4 T cells were modest. We therefore next investigated the TCR Vβ distribution of these cells, where a strongly biased Vβ distribution would be consistent with a narrowly antigen-driven expansion. Surprisingly, the DEM CD4 T cells displayed a diverse Vβ distribution very similar to the overall CD4 T cell compartment (Figure 6D). Taken together, these results support a model where a broad array of antigens support the generation of diverse DEM CD4 T cells in the context of the innate immune activation milieu that persists in chronically HIV-1 infected individuals.

## Discussion

In the present study, we aimed to better understand persistent immune activation in the CD4 T cell compartment of HIV-1 infected patients in rural Uganda, and to investigate relationships with possible causes of such activation. The results suggest that pathologically activated CD4 T cells have a Ki67+ effector memory phenotype, and because they are activated despite their expression of PD-1, and correlate inversely with FoxP3+ Treg levels, we use the term deregulated effector memory (DEM) cells to describe them.

Monocyte activation in response to microbial material results in the release of sCD14, which is seen at elevated levels in infected subjects [12], [42]. We found that sCD14 correlated inversely with CD4 counts and directly with DEM CD4 T cell levels. sCD14 also correlated positively, albeit weakly, with viral load. Notably, however, groups with high and low levels of sCD14 did not differ in their pace of disease progression during clinical follow-up. IL-6 is released in response to activation of innate cells, including monocytes, and displayed correlations with CD4 counts and DEM CD4 T cells similar to those of sCD14. Also in similarity with sCD14, the correlation with viral load was very weak. However, in contrast to sCD14, patients with high and low levels of IL-6 differed in their rate of progression to AIDS during clinical follow-up. One might therefore speculate that IL-6 is more likely than sCD14 to indicate pathogenic processes in the innate immune system during chronic untreated HIV-1 infection. Treg levels correlated inversely with DEM CD4 T cells, but showed no correlation with CD4 count, viral load, IL-6 or sCD14. Changes in Treg percentages thus appear to be primarily associated with the activated DEM CD4 T cell expansion, and less so to the other measures of disease.

We observed that the DEM CD4 T cell phenotype can be driven *in vitro* by stimulation of healthy donor PBMC with recall antigens for six days. In particular, stimulation with CMV and Candida antigens resulted in effective expansion of such cells. In addition, there was an enrichment of HIV-specific and CMV-specific cells in the DEM CD4 T cell subset in patients. These observations support a model where the DEM cell expansion is at least partly antigen-driven. We were therefore surprised to find that the TCR Vβ distribution was not significantly different between these cells and the rest of the CD4 compartment in HIV-1 infected subjects. If the DEM CD4 T cells were strongly driven by a few antigens, then one would expect a biased Vβ distribution. Since this was not the case, it seems likely that the expanded DEM CD4 T cells are specific for a diverse array of antigens from HIV, CMV, and other pathogens. We therefore suggest a model in which the accumulation of DEM CD4 T cells is the result of antigen-driven expansion under conditions when the immune system is exposed to microbial material, including components of HIV, driving innate immune activation.

The correlation between levels of IL-6 and DEM CD4 T cells, albeit weak, initially suggested that this cytokine might be involved in the generation or maintenance of these cells [43]. HIV-1 infection has long been associated with increased levels of IL-6 [44], [45], and IL-6 can be produced by monocytes in response to both LPS and the HIV-1 accessory protein Vpr [46], suggesting that both viral and bacterial antigens could contribute to the increased levels of this inflammatory cytokine. In the present study, blocking of IL-6 and IL-6R or addition of exogenous IL-6 to cultures had no measureable effect (data not shown). This does not contradict a possible role of IL-6 during long-term exposure. Another factor of interest, according to the hypothesis that excessive TLR7/9 signaling in response to HIV RNA underlies persistent immune activation, is type I IFNs [18], [19], [20]. However, similar to the results with IL-6, we observed no effect by addition of IFNα in cultures (data not shown). Of note, IFNα treatment of HIV patients on successful ART co-infected with hepatitis C virus was previously observed to reduce immune activation [47]. Similarly, we observed no effect on DEM cell expansion by adding whole inactivated HIV to short-term *in vitro* cultures (data not shown). Again, these observations do not contradict a pathogenic contribution of type I IFNs *in vivo*, in particular as a co-factor working together with other causes of activation.

In a previously published longitudinal study based on this Ugandan cohort, soluble markers of innate immune activation in plasma did not change over the course of HIV disease progression [48]. The relationships between microbial translocation, chronic activation of innate and adaptive immune cells, levels of cytokines and other markers in plasma, and HIV disease progression are thus very complex. However, the present study supports an association between innate immune activation and expansion of the DEM CD4 T cells. This CD4 T cell phenotype may identify the cells, which are activated by chronic antigen stimulation in the context of innate immune system activation by microbial components. Such material may be derived from microbial translocation, as well as from HIV virions. Here, levels of DEM CD4 T cells correlated directly to HIV viral load, possibly supporting a role for viral proteins or RNA in the expansion of these cells.

The DEM CD4 T cells display a CD45RO+CCR7- effector memory phenotype, and they express Ki67 at a higher rate than the overall CD4 T cell compartment. The relatively frequent expression of Ki67 suggests that this is an actively proliferating cell subset despite the expression of PD-1, which is a negative regulator of TCR signaling. The question arises as to what extent PD-1 is a strong inhibitory receptor in CD4 T cells *in vivo*. One possibility is that the negative signals mediated by PD-1 are counter balanced by co-stimulation via CD28 [49].

The data presented in this paper are consistent with a model where continuous exposure to microbial material, including components of HIV, creates conditions permissive to the expansion pathologically activated CD4 T cells with the DEM phenotype. The accumulation of DEM CD4 T cells may result from antigen-driven expansion under conditions of persistent innate immune activation. Future studies to examine the mediators of pathogenic T cell activation are needed to allow the development of targeted therapies to alleviate activation and, possibly, slow disease progression.
